# Factors associated with early childhood stunted growth in a 2012–2015 birth cohort monitored in the rural Msambweni area of coastal Kenya: a cross-sectional study

**DOI:** 10.1186/s12887-020-02110-z

**Published:** 2020-05-12

**Authors:** Shanique Martin, Francis Mutuku, Julia Sessions, Justin Lee, Dunstan Mukoko, Indu Malhotra, Charles H. King, A. Desiree LaBeaud

**Affiliations:** 1 Stanford University School of Medicine, 291 Campus Drive, Stanford, CA 94305 USA; 2 Technical University of Mombasa, Mombasa, Kenya; 3 Vector-Borne Diseases Control Unit, Ministry of Health, Nairobi, Kenya; 4 Case Western Reserve University, Center for Global Health and Diseases, Cleveland, OH USA

**Keywords:** Child growth, Malnutrition, Parasitic infections, Global health

## Abstract

**Background:**

Chronic malnutrition, often measured as stunted growth, is an understudied global health problem. Though poor nutritional intake has been linked to stunted growth, there is evidence suggesting environmental exposures may have a significant role in its occurrence. Here, we characterize the non-nutritional prenatal and postnatal factors that contribute to early childhood stunted growth in rural coastal Kenya.

**Methods:**

Overall, 232 women and 244 children from a 2012–2015 maternal-child cohort in Msambweni, Kenya were included. Women were tested for parasitic infections during the prenatal period and at the time of delivery. Children were tested for parasitic infections and assessed for stunted growth using height-for-age Z-scores (HAZ) at 6-month intervals after birth. Socioeconomic status (SES) was evaluated using both a simplified water, asset, maternal education, and income (WAMI) index and a principal component analysis (PCA) asset score. Multivariate logistic regression analysis was used to determine the relative influence of prenatal and postnatal factors on the occurrence of stunted growth.

**Results:**

Of the 244 children (ages 6–37 months), 60 (25%) were stunted at the study endpoint. 179 mothers (77%) had at least one parasitic infection during pregnancy and 94 children (38%) had at least one parasitic infection during the study period. There was no significant association between maternal parasitic infection and child stunted growth (*p* = 1.00). SES as determined using the WAMI index was not associated with HAZ in linear regression analysis (*p* = 0.307), however, the PCA asset score was (*p* = 0.048). Multivariate logistic regression analysis identified low birth weight (AOR: 3.24, 95% CI: [1.38, 7.57]) and child parasitic infectious disease burden (AOR: 1.41, 95% CI: [1.05, 1.95]) as independent predictors of stunted growth, though no significant association was identified with PCA asset score (AOR: 0.98, 95% CI: [0.88, 1.10]).

**Conclusions:**

Stunted growth remains highly prevalent in rural Kenya, with low birth weight and child parasitic infectious disease burden demonstrated to be significantly associated with this indicator of chronic malnutrition. These results emphasize the multifaceted nature of stunted growth and the need to address both the prenatal and postnatal environmental factors that contribute to this problem.

## Background

Chronic malnutrition affects an estimated 165 million children younger than 5 years worldwide [1]. Though the global prevalence is decreasing, this problem remains an important topic of investigation given its known effects on child mortality and contributions to the occurrence of chronic and irreversible morbidity if left untreated [1–3]. Although it is a worldwide health problem, the prevalence remains highest in low-income countries [4, 5], with an estimated prevalence of 30% in Kenya [6–8]. Height-for-age Z score (HAZ), weight-for-age Z score (WAZ) and weight-for-height Z-score (WHZ), which reflect deviations from statistical growth norms, are widely accepted as indicators of malnutrition. Stunted growth, defined as HAZ < − 2, is often used as an indicator of chronic malnutrition and has been associated with poor health outcomes [9].

Though inadequate food intake during childhood is one established cause of acute malnutrition and diminished linear growth, chronic malnutrition and stunted growth may also be the result of a variety of other environmental factors. Both childhood exposure to infectious disease and household socioeconomic status (SES) have been demonstrated to correlate with stunted growth [10]. Infant low birth weight, (< 2500 g), which has known links to maternal tobacco use, undernutrition, and anemia, has also been demonstrated to predict stunted growth in childhood, indicating that prenatal exposures may contribute chronic malnutrition [11–13]. In rural Kenya, given the high prevalence of acute and chronic infections, infectious disease burden is an important variable that must be considered as a contributor to the growth of a child. There is some evidence to suggest that early childhood growth may be influenced by child infection with parasitic pathogens, however, the role this plays in the context of maternal prenatal parasitic infection has not been well studied [14–17].

The degree to which environmental factors influence linear growth and malnutrition may be unique to each population of interest. In the present study, we aimed to measure the prevalence of early childhood stunted growth in rural coastal Kenya and characterize the non-nutritional prenatal and postnatal factors of greatest influence on this indicator of chronic malnutrition.

## Methods

This study analyzed data from a cohort of mother-child pairs enrolled in an investigational study on prenatal parasitic infections and vaccine response conducted at the Msambweni District Hospital in rural coastal Kenya [18, 19]. This study was approved by the Internal Review Boards of Stanford University (IRB# 31468), Kenyatta National Hospital/University of Nairobi in Kenya (P85/03/2013), and Case Western Reserve University (IRB# 01–13-13).

### Study population

Pregnant women who provided informed consent for themselves and their child/children were enrolled. The women agreed to receive prenatal and postnatal care at the Msambweni District Hospital as well as to bring their child/children to Msambweni District Hospital for study follow-up visits. Of the 596 mother-child pairs enrolled in the longitudinal vaccine response study, 332 had scheduled follow-up visits within the 6-week sub-study period. Overall, 232 women and 244 children (including 6 twin pairs) were included in the study presented here. Attendance to a study follow-up visit in the 6-weeks between June and July 2016, at which time child parasitic infection testing was performed and the household SES survey was administered, was required for maternal-child inclusion in this study.

### Data collection: environmental factors of interest

Upon enrollment, the mothers underwent testing for parasitic infections during the prenatal period, as well as at delivery, through blood, urine, and stool testing. They were tested for malaria, *Schistosoma haematobium, Entamoeba histolytica, Giardia*, lymphatic filariasis, and soil-transmitted helminths (STH), including hookworm*, Strongyloides stercoralis, Ascaris lumbricoides,* and *Trichuris trichiura*. Blood smear as well as polymerase chain reaction/ligase detection reaction (PCR/LDR) performed using a red blood cell pellet were used to determine active malarial infection. Either a positive blood smear or a positive PCR/LDR was considered evidence of active malarial infection. Fresh urine samples were filtered then microscopically screened for *S. haematobium* eggs and plasma was tested for anti-soluble worm adult protein (SWAP) IgG4. The presence of any number of eggs or IgG4 positivity for SWAP was considered positive for *S. haematobium* infection*.* The Ritchie Method was used to evaluate stool for ova and larvae of any STH as well as *Giardia* and *E. histolytica* [20]. Lymphatic filariasis infection was assessed by ELISA detection of *Brugia malayi* antigen (BMA)-specific IgG4 antibodies.

From the time of delivery, the children underwent general physical examination in addition to parasitic infection testing through blood, urine, and stool examination at 6 weeks, 10 weeks and 6 months of age, as well as at each subsequent 6-month age increment during the study period. Children were screened for the aforementioned parasitic infections, except for lymphatic filariasis. The screen for *S. haematobium* was performed for all children who were old enough to provide a urine sample. All subjects found to be positive for any parasitic infection were provided with the appropriate treatment.

At the final study follow-up visit, occurring between June to July 2016, the mothers (or the primary guardian of the child) completed an SES survey (See Supplementary Table 1, Additional File 1). This survey was established by the Malnutrition and Enteric Infections: Consequences for Child Health and Development (MAL-ED) study which led to the development of the WAMI index, a simplified composite SES score consisting of four components: 1) access to improved water and sanitation, 2) ownership of eight selected assets, 3) maternal education, and 4) monthly household income [21]. In its development, the WAMI index was evaluated against child HAZ across 8 countries, demonstrating good linear fit and validating its use for SES comparisons between developing countries [21]. With permission and guidance from the original developers, an adapted version of the WAMI index survey was designed to assess maternal characteristics, including age, education, and obstetric history, as well as household size, water access, sanitation facility, assets, and home characteristics. The 84-question survey was administered verbally by a single trained staff member in Kiswahili or the preferred tribal dialect of the mother/primary guardian. The study follow-up period was defined as the time between birth of the enrolled child and the final study follow-up visit, reported here as the child’s age at that visit.

### Data collection: primary outcome

Trained clinical staff recorded standardized anthropometric measurements of the children at each visit, including length/height (cm), weight (kg), and head circumference (cm). Recumbent length was measured for children less than 2 years of age, and standing height was measured for children 2 years of age and older, both to the nearest 0.1 cm. Weight was recorded to the nearest 0.1 kg using a digital scale. Anthropometric measurements were obtained twice for each child at every visit, with each measurement performed by separate members of the clinical staff then compared in real-time for consistency. Any discrepancies were resolved by immediate re-measurement.

The anthropometric measurements of the children were transformed into age and gender-specific Z-scores using the WHO Anthro software (WHO, Geneva, Switzerland) based on the WHO Child Growth Standards for normal child growth across the world, from birth until the age of 5 [22]. HAZ, WAZ and WHZ scores were calculated for each time point considered in this study. Using the global median, the three categories of malnutrition – stunting, underweight, and wasting – were defined as greater than 2 standard deviations (SD) below the global median for height/length-for-age, weight-for-age, and weight-for-height respectively, corresponding to Z scores < − 2. The primary outcome of this study was stunted growth at the final follow-up visit, defined as HAZ < -2.

### Statistical analysis

WAMI index SES scores were calculated for each child’s household using the method previously defined by Psaki et al. Principal component analysis (PCA) was used to stratify the study population by SES using maternal and household characteristics from the present SES survey. Household use of toilet paper was not included in the PCA as in this study population lack of toilet paper use was determined by religious practice, predominantly Islam, and thus was not considered a representative indicator of SES. Twin pair data were evaluated independently with the exception of maternal prenatal parasitic infection data and household SES survey data, which were necessarily shared for twin pairs.

Associations between independent variables and the primary outcome were assessed using bivariate analysis, with Student’s t-testing used for continuous data and chi-square or Fisher’s exact test used for categorical data. A multivariate logistic regression model explaining variations in the occurrence of stunted growth was created using independent variables determined to have a trend towards significant association with current stunted growth on bivariate analysis. As the 6-month periodic cohort follow-up adherence was not 100% for all study participants, a subset analysis was performed on the cohort of 77 children who did not miss any scheduled follow-up visits from birth to 24 months. Statistical analyses were performed using R (Version 3.3.1, R Foundation for Statistical Computing, Vienna, Austria).

## Results

### Participant characteristics

Of the 244 child participants, 101 (41%) were male and the ages at the study endpoint ranged from 6 to 37 months of age, with a mean age of 20.5 months (Table 1). At the study endpoint, 60 children (25%) were stunted and retrospective analysis showed that 131 (54%) were stunted during at least one prior biannual visit. There was no difference in the gender distribution among stunted children and the overall study population. At the study endpoint, 17 children (7%) were underweight (WAZ < -2) and 11 (5%) were wasted (WHZ < -2). Participant stunted growth was associated with concurrent underweight classification (*p* < 0.01), though no association was seen with wasting. Children with low birth weight (< 2500 g) were more likely to be stunted at the study endpoint (*p* = 0.01).

**Table 1:** Characteristics of the study subjects

Characteristics	Total Cohort (*n* = 244 (100%))	Linear Growth at Study Endpoint		*p*-value
		Normal (*n* = 184 (75%))	Stunted (*n* = 60 (25%))	
Age, mo - mean ± SD	20.51 ± 7.01	20.24 ± 7.10	21.32 ± 6.71	0.30^a^
Gender - n (%)				
Male	101 (41.39)	76 (41.30)	25 (41.67)	1.00^c^
Female	143 (58.61)	108 (58.70)	35 (58.33)	1.00^c^
Maternal Pregnancy History - mean ± SD				
Age at first Pregnancy	20.18 ± 3.81	20.27 ± 3.44	19.89 ± 4.83	0.52^a^
Gravida	4.14 ± 2.44	4.05 ± 2.31	4.42 ± 2.81	0.31^a^
Parity	3.16 ± 1.94	3.07 ± 1.69	3.45 ± 2.55	0.18^a^
Additional Pregnancy^d^ - n (%)	12 (4.92)	8 (4.35)	4 (6.67)	0.71^b^
Maternal Infection Burden during Pregnancy - n (%)				
Prenatal				
None	65 (26.64)	49 (26.63)	16 (26.67)	1.00^c^
1 Infection	91 (37.29)	65 (35.33)	26 (43.33)	0.34^c^
2 Infections	63 (25.82)	49 (26.63)	14 (23.33)	0.74^c^
3+ Infections	25 (10.25)	21 (11.41)	4 (6.67)	0.42^b^
At Delivery				
None	142 (58.20)	109 (59.24)	33 (55.00)	0.67^c^
1 Infection	74 (30.33)	55 (29.89)	19 (31.67)	0.92^c^
2 Infections	24 (9.83)	19 (10.33)	5 (8.33)	0.84^b^
3+ Infections	4 (1.64)	1 (0.54)	3 (5.00)	0.08^b^
Birth weight, g – mean ± SD	3007 ± 463	3081 ± 435	2782 ± 475	< 0.001^a^
Low Birth Weight (< 2500 g)	26 (10.66)	14 (7.61)	12 (20.00)	0.01^c^
Other Nutritional Proxies - n (%)				
Underweight (WAZ < -2)	17 (6.97)	5 (2.72)	12 (20.00)	< 0.001^c^
Wasted (WHZ < -2)	11 (4.51)	6 (3.26)	5 (8.33)	0.20^c^
Child Infection Burden - n (%)				
Malaria				0.02^b^
0 Times	209 (85.65)	162 (88.04)	47 (78.33)	
1 Time	26 (10.66)	14 (7.61)	12 (20.00)	
2+ Times	9 (3.69)	8 (4.35)	1 (1.67)	
Hookworm				0.11^b^
0 Times	217 (88.93)	168 (91.30)	49 (81.67)	
1 Time	23 (9.43)	14 (7.61)	9 (15.00)	
2+ Times	4 (1.64)	2 (1.09)	2 (3.33)	
*Trichuris*				0.26^b^
0 Times	233 (95.49)	178 (96.74)	55 (91.67)	
1 Time	9 (3.69)	5 (2.72)	4 (6.67)	
2+ Times	2 (0.82)	1 (0.54)	1 (1.67)	
*Ascaris*				0.07^b^
0 Times	235 (96.31)	180 (97.83)	55 (91.67)	
1 Time	9 (3.69)	4 (2.17)	5 (8.33)	
*Giardia*				0.64^b^
0 Times	218 (89.34)	164 (89.13)	54 (90.00)	
1 Time	24 (9.84)	19 (10.33)	5 (8.33)	
2+ Times	2 (0.82)	1 (0.54)	1 (1.67)	
*Strongyloides*				0.30^b^
0 Times	241 (98.77)	183 (99.46)	58 (96.67)	
1 Time	3 (1.23)	1 (0.54)	2 (3.33)	
*Entamoeba*				0.57^b^
0 Times	235 (96.31)	176 (95.65)	59 (98.33)	
1 Time	9 (3.69)	8 (4.35)	1 (1.67)	
Any Infection	94 (38.52)	63 (34.24)	31 (51.67)	0.02^c^
Total Infections- mean ± SD	0.57 ± 0.96	0.49 ± 0.95	0.82 ± 0.98	0.02^a^

^a^ = t-test; ^b^ = Fisher’s exact test; ^c^ = Chi-square test; ^d^Additional pregnancy after the birth of the enrolled child

### Stunted growth and infection

Of the children stunted at the study endpoint, 179 (73%) had mothers with at least one parasitic infection during the prenatal period and 102 (42%) had mothers with at least one parasitic infection at delivery. The most common prenatal infection was malaria, with 96 mothers (41%) infected at a prenatal visit and 8 mothers remaining infected at the time of delivery. Ninety-four children (39%) had at least one infection during the study period. Bivariate analysis demonstrated no significant association between maternal prenatal parasitic infection status, maternal delivery parasitic infection status, or maternal obstetric history and child stunted growth (Table 1).

**Fig. 1:**
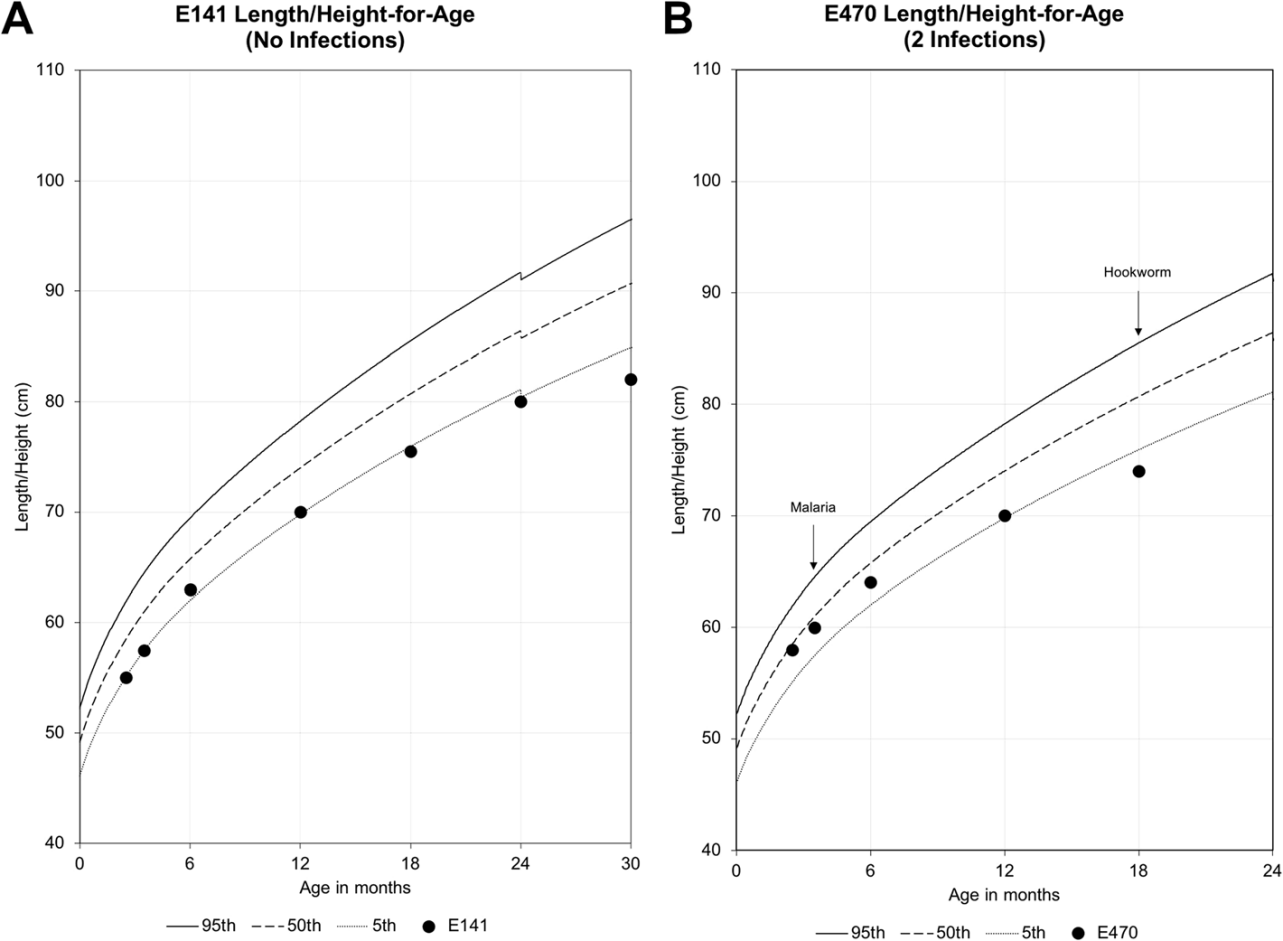
Height-for-age growth trends for two study participants who were stunted at the study end point. **a** Participant E141 started early infancy with growth along the 5th percentile until 18 months of age, after which there is a steady decrease in growth below the 5th percentile. This participant had no childhood infection history. **b** Participant E470 started early infancy with growth along the 50th percentile, was infected with malaria at 10 weeks of age, after which there is a steady decrease in growth to the 5th percentile. The child was subsequently infected with hookworm at 18 months at which point her growth was below the 5th percentile. Both participants E141 and E470 had normal birth weights

Malaria was the most common childhood parasitic infection detected, with 35 children (14%) found to be infected during the study period, 9 of whom were positive at two or more separate visits. As a parasitic group, STH were the most prevalent infection in the children, with 73 (30%) infected with at least 1 STH in the study period. *S. haematobium* was removed in the final analysis due to a high proportion of children being unable to provide adequate urine samples throughout the study. Overall, early childhood infectious disease burden (malaria, *Giardia*, *Entamoeba,* or any STH infection) was significantly correlated with current stunted growth (*p* = 0.02) though this significance was primarily driven by malaria infection (Table 1). Childhood parasitic infection was associated with lower HAZ and in some individual cases the diagnosis of a parasitic infection corresponded with a measured decrease in HAZ (Fig. 1b). This temporal relationship was not true for all children found to be stunted in this study as there were children for whom no change in linear growth was seen in the time immediately following an infection. There were also children with persistent low HAZ – at or below the 5th percentile (HAZ < − 1.65) – since birth in the absence of any diagnosed parasitic infection during childhood (Fig. 1a).

**Fig. 2:**
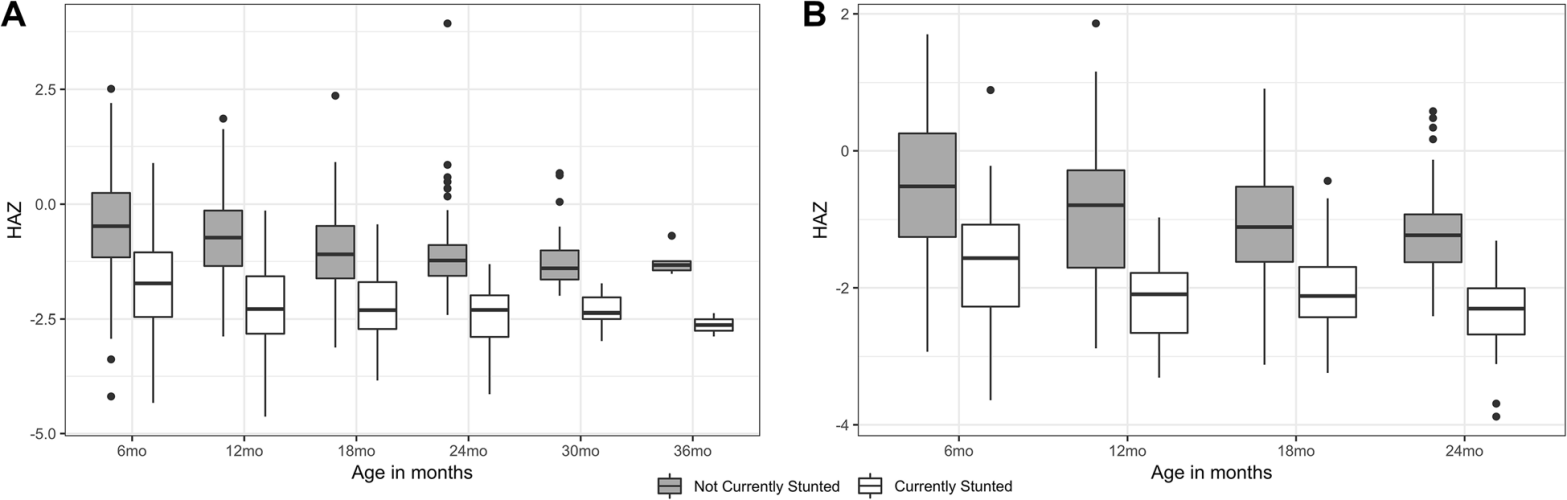
HAZ at 6-month intervals for children with normal HAZ at the study end point and those stunted at the study end point. **a** Mean HAZ from 6 to 36 months of age for all study participants (n = 244). **b** Mean HAZ at 6-month intervals for the subset of participants (*n* = 77) with complete anthropometric data from 6 to 24 months of age

The largest change in the prevalence of stunted growth (+ 7.5%) occurred between 6 months and 12 months of age with a peak prevalence of 26.6% at 18 months of age. Similar trends in the prevalence of stunted growth across age groups were seen in the subset of 77 children for whom all anthropometric data from 6 months to 24 months of age was available. In both the overall study population and the 77 subset, there was a trend towards a decrease in HAZ over time; however, on retrospective analysis, children who were stunted at the study endpoint had on average a lower HAZ throughout the entirety of the study compared to children with normal HAZ (Fig. 2).

### Stunted growth and SES

**Fig. 3:**
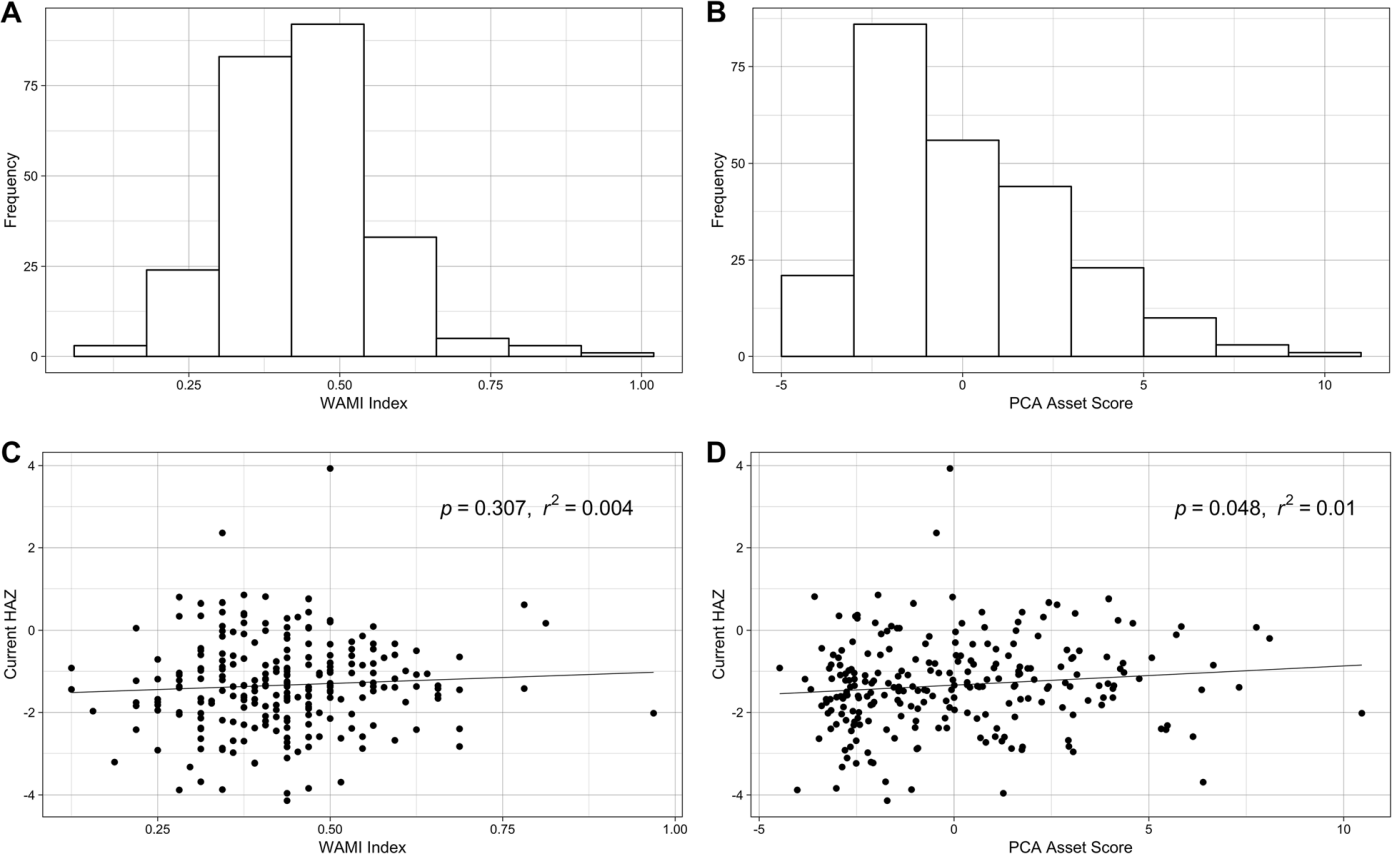
SES as determined by PCA and WAMI. **a** Distribution of household WAMI index for all participants. **b** Distribution of household PCA asset score for all participants. **c** Linear regression of child HAZ and WAMI index at the study end point. **d** Linear regression of child HAZ and PCA asset score at the study end point

**Fig. 4:**
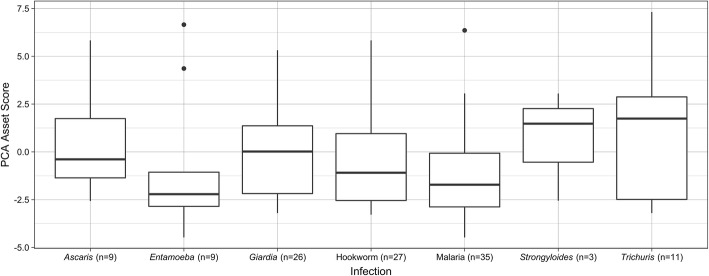
Frequency of PCA asset scores by type of childhood parasitic infections

WAMI index scores were calculated using the methodology described by Psaki et al. As a simplified SES indicator, the WAMI index did not correlate with current HAZ (Fig. 3c). Principal component analysis using the present study’s SES survey data was used as a secondary method to assess SES of the study participants. The combination of low frequency options within each question category was limited to those of similar economic value in order to best preserve the ability of the PCA method to detect SES variation within this small, rural population (Table 2). There were 58 resulting variables from which 55 were chosen due to the removal of two variables with low frequencies (domestic worker (1.6%) and computer (1.2%)) and one with a high frequency (ownership of a mat or a bench (98%)). Variables that were predicted to be associated with lower SES had negative factor scores, including having a greater ratio of people to rooms in a household, having mud walls, and not having a toilet facility. In cases of a missing response, a factor score of zero was assigned for those questions. A PCA asset score was calculated for each household using the summation of the factor scores and this asset score correlated with current HAZ (Fig. 3d, *p* = 0.048). Low PCA asset score was not associated with low current WAZ or WHZ. There was however, a noted association between PCA asset score and type of parasitic infection, with the majority of the tested types of parasitic infections occurring predominantly in children with household PCA asset scores less than or equal to zero (Fig. 4).

**Table 2:** Socioeconomic status PCA variables and factor scores

Variable Description	Mean/Frequency	Factor Score
**Maternal Education** – mean ± SD		
School	5.60 ± 4.04	0.161
Religious School	2.80 ± 3.25	0.034
**Household** – mean ± SD		
Number of People per Room	2.78 ± 1.05	− 0.099
**Cooking** – n(%)		
Fuel		
Open Fire	235 (96.3)	−0.092
Kerosene	7 (2.9)	0.065
Unspecified	2 (0.8)	0.000
Location		
Inside House = yes	220 (90.2)	0.027
**Home** – n(%)		
Floor Material		
Wood/Ceramic/Vinyl	3 (1.2)	0.026
Cement/Concrete	113 (46.3)	0.274
Earth/Sand/Clay/Mud/Dung	127 (52.0)	−0.277
Unspecified	1 (0.4)	0.000
Roof Material		
Metal/Tiles	39 (16.0)	0.141
No Roof/Other	95 (38.9)	0.041
Thatch Roof	110 (45.1)	−0.144
Exterior Wall		
Stone/Other	10 (4.1)	0.001
Cement/Concrete	114 (46.7)	0.284
Mud	120 (49.2)	−0.284
**Drinking Water Source** - n (%)		
Water Collection		
Unprotected Dug Well/Surface Water	8 (3.3)	−0.061
Protected Well	85 (34.8)	−0.11
Tube Well/Bore Hole	65 (26.6)	−0.068
Public Tap/Stand Pipe	83 (34.0)	0.2
Piped Into Dwelling/Yard/Plot	3 (1.2)	−0.012
Continuous Water = yes	146 (59.8)	− 0.1
Pay for Water = yes	132 (54.1)	0.087
Water in own yard/plot = yes	80 (32.8)	0.107
Treat Water = yes	172 (70.5)	0.113
**Other Water Source** - n (%)		
Water Collection		
Surface Water/Other	32 (13.1)	−0.068
Protected Spring/Rain Water/ Unprotected Dug Well	10 (4.1)	−0.038
Protected Well	84 (34.4)	−0.081
Tube Well/Bore Hole	45 (18.4)	−0.048
Public Tap/Stand Pipe	69 (28.3)	0.201
Piped into Dwelling/Yard/Plot	3 (1.2)	−0.012
Unspecified	1 (0.4)	0.000
**Sanitation** - n (%)		
Toilet Facility		
No Facility	120 (49.2)	−0.202
Pit Latrine without Slab	81 (33.2)	0.053
Flush to somewhere else	33 (13.5)	0.182
Flush/Pour-Flush/Pit Latrine with Slab/ Composting	10 (4.1)	0.068
Shared Toilet = yes	141 (57.8)	−0.129
**Assets** - n (%)		
Electricity	78 (32.0)	0.199
Iron	49 (20.1)	0.212
Mattress	234 (95.9)	0.06
Sofa	33 (13.5)	0.181
Cupboard	11 (4.5)	0.156
Table	206 (84.4)	0.077
Electric Fan	10 (4.1)	0.138
Radio	125 (51.2)	0.145
Television	41 (16.8)	0.212
Mobile Phone	210 (86.1)	0.091
Fridge	10 (4.1)	0.164
Watch or Clock	24 (9.8)	0.125
Motorized Vehicle	20 (8.2)	0.087
Bicycle	97 (39.8)	0.023
Bank Account	60 (24.6)	0.176
Agricultural Land	174 (71.3)	−0.066
Coconut Trees	149 (61.1)	−0.037
Acres of Land – mean ± SD	2.40 ± 3.34	−0.012
Cows/Goats	82 (33.6)	0.072
Chickens/Ducks	144 (59.0)	−0.018

### Multivariate model

Multivariate logistic regression analysis was used to assess the impact of childhood factors on stunted growth in this population (Table 3). Only variables with *p*-values less than 0.1 in bivariate analysis were included. The adjusted odds of stunted growth at the study end point were 3.24-fold higher in children with low birth weight (95% CI: [1.38, 7.57]) and increased by 1.41 with each parasitic infection occurring during childhood (95% CI: [1.05, 1.95]). SES, determined using PCA asset score, was not a significant predictor of stunted growth in this multivariate model (AOR: 0.98, 95% CI: [0.88, 1.10]). Internal validation testing of the regression model yielded Hosmer-Lemeshow statistic of 0.9697 indicating good model fit, and a C-statistic or AUC of 0.6511 indicating good model discrimination.

**Table 3:** Multivariate logistic regression results for stunted growth at the study end-point

	Coefficient (***β***)	SE	t-value	p-value	Adjusted Odds Ratio	95% CI
Intercept	−1.49	0.20	−7.50	< 0.01	0.23	0.15–0.33
Low Birth Weight	1.18	0.43	2.73	0.01	3.24	1.38–7.57
Number of Parasitic Infections	0.34	0.16	2.17	0.03	1.41	1.05–1.95
PCA Asset Score	−0.02	0.06	−0.28	0.78	0.98	0.88–1.10

## Discussion

These results demonstrate that the prevalence of early childhood stunted growth in rural coastal Kenya remains high, though it is 5% lower than the 2012 WHO estimates and lower than the prevalence reported by other recent independent studies in the nation [6–8]. There were no significant differences in maternal pregnancy or prenatal infection history between the normal and stunted children. Interestingly, children who were stunted at the end of the study had, on average, a lower HAZ at each 6-month interval from 6 to 36 months of age when compared to children who were not stunted at the study endpoint. This held true for the subset of 77 children for which anthropometric data were available for every 6-month interval from 6 months to 24 months of age. In this group, the mean differences in HAZ were significantly different between the stunted and normal children at every 6-month interval since early infancy (See Supplementary Table 2, Additional File 2). This, in addition to our observation of children with persistently low HAZ since birth, further emphasizes the importance of assessing prenatal and early infancy environmental exposures.

In this population, maternal prenatal parasitic infection did not explain the predilection to having low HAZ in early infancy and among the early infancy variables studied, birth weight was the only significant predictor of stunted growth. Intrauterine fetal growth restriction resulting in low birth weight is known to be influenced by maternal health and environmental exposures during pregnancy. These exposures, including tobacco use and poor nutrition, were not directly measured in this study yet the demonstrated significant association between low birth weight and stunted growth in childhood indicates a need for further investigation and characterization of these unmeasured prenatal environmental exposures in this community. Though similar associations between low birth weight and stunted growth have been demonstrated in pediatric populations of developing countries [23, 24], this in combination with the observed association of stunted growth with childhood parasitic infectious disease burden, has not previously been reported in the coastal Kenya setting. In this study, both low birth weight and childhood parasitic infectious disease burden were independent predictors of stunted growth, and the observed lower birth weight and consistently lower HAZ throughout childhood for children stunted at the study end point suggests that there are additional environmental factors contributing to malnutrition present during the prenatal period and early infancy, to which childhood parasitic infection burden may have an additive effect.

SES, when determined using the PCA method, correlated with current HAZ, though our inability to assess SES at the time of prenatal enrollment in the study remains a limitation in accurately assessing its relationship with low birth weight and low HAZ in early infancy. The WAMI index was not sufficient to characterize wealth distribution within this population, as seen by the lack of its correlation with child HAZ at the time of the survey administration (HAZ was the outcome that was used to validate the WAMI index in its development [21]). Even so, the accuracy of the income estimates used in the calculation of the WAMI index remains in question. Many of the surveyed mothers expressed uncertainty about their households’ monthly income as they were not the primary source of income for their families. This introduces the possibility that SES measures utilizing reported income may be less accurate in populations where the survey respondents are not primary wage earners in their households.

Though the PCA method, which excluded self-reported income, was superior to WAMI for characterizing SES in this population, it is a complex method that requires large data inputs to stratify a population. In this population, low frequencies of piped water, an asset typically associated with wealth in rural populations, led to an assigned negative factor score (Factor Score = − 0.012; Table 2), demonstrating an additional limitation of the PCA method. Even so, in this case, the absolute value of the factor was low thus its contribution to overall PCA asset score was not substantial.

Here, we have identified maternal prenatal health, measured as child low birth weight, and child parasitic infectious disease burden as variables with significant influence on the occurrence of early childhood stunted growth. This study is, however, not without limitations. In choosing to characterize only the non-nutritional causes of stunted growth in this population, child nutritional intake is a confounder that was not examined in this study. We predict that this factor may play a role in the occurrence of stunted growth in this community, given our research group has previously reported a lifetime average blood hemoglobin concentration in the anemic range (< 11 g/dL) in 95% of this pediatric population [10], indicating chronic micronutrient deficiency. Even so, such a high prevalence of anemia in this population is unlikely to explain the observed variation in the occurrence of stunted growth.

This study is also limited by the loss to follow-up rate of 30%, with only 232 of the 332 mothers scheduled bring their child for follow-up, returning to the clinic in the summer 2016 observation period. The reasons for loss to follow-up are unknown, however, attempts made to contact maternal-child pairs indicate that family migration to a different community further away from the study site is the predominant reason for the missed follow-up visit. Even so, the identification of independent associations between child low birth weight and parasitic infectious disease burden with current stunted growth provides additional insight into the non-nutritional causes of stunted growth in this rural coastal Kenyan community. These results suggest that interventions in this population targeting the improvement of maternal prenatal health and reducing childhood exposure to parasitic infection may lead to improved child growth outcomes.

## Conclusions

Our findings demonstrate that low birth weight along with childhood infectious disease burden may be non-nutritional risk factors for stunted growth and chronic malnutrition in a rural Kenyan pediatric population. Previous studies have reported that maternal prenatal parasitic infections can affect childhood immunologic response to vaccines, but our study found no association between prenatal parasitic infections and early childhood growth [25]. Even so, the high childhood parasitic infection burden observed in this population suggests that additional investigation into the potential effects on childhood susceptibility to infections may be warranted [25, 26]. The prevalence of child chronic malnutrition, as measured by stunted growth, remains elevated in rural Kenya. Though low birth weight and parasitic infection burden have been shown to be contributors to this problem, improved characterization of SES and other environmental risk factors of malnutrition present in the prenatal and postnatal period is still needed. In rural communities of developing countries, where the prevalence of stunted growth remains high, continued evaluation of these non-nutritional risks will allow for the development of targeted efforts to combat this global child health problem.

## Supplementary information

**Additional File 1.** Socioeconomic Status Survey. The 84-question socioeconomic status and family planning survey used to determine SES using WAMI index and PCA asset score. Adapted from Psaki et al.

**Additional File 2.** Standardized mean difference of HAZ at 6-month intervals, by stunting status, for all study participants and for the subset of 77 study participants with complete anthropometric data from 6 to 24 months of age.

## Data Availability

The datasets used and/or analyzed during the current study are available from the corresponding author on reasonable request.

## Abbreviations

SES: Socioeconomic status
WAZ: Weight-for-age Z score
WHZ: Weight-for-height Z-score
HAZ: Height-for-age Z-score
STH: Soil-transmitted helminths
SWAP: *Schistosoma* worm adult protein
PCR: Polymerase chain reaction
LDR: Ligase detection reaction
SD: Standard deviation
PCA: Principal component analysis
MAL-ED: Malnutrition and Enteric Infections: Consequences for Child Health and Development
WAMI: Water, asset, maternal education, and income
AUC: Area under the curve
