# Supplementary material for: Factors associated with early childhood stunted growth in a 2012–2015 birth cohort monitored in the rural Msambweni area of coastal Kenya: a cross-sectional study
Source: BMC Pediatr. 2020 May 12;20:208. doi: 10.1186/s12887-020-02110-z (PMC7216696; doi:10.1186/s12887-020-02110-z)
Supplement: Supplementary file 1 — Additional File 1. Socioeconomic Status Survey. The 84-question socioeconomic status and family planning survey used to determine SES using WAMI index and PCA asset score. Adapted from Psaki et al. [file 12887_2020_2110_MOESM1_ESM.docx]

**Supplemental Table 1, Additional File 1:** Socioeconomic Status Survey (adapted from Psaki et al)

| **DEMOGRAPHIC QUESTIONS** | | | |
| --- | --- | --- | --- |
| **#** | **Question** | **Code** | **Response** |
| *These questions are intended for the mother of the child. Complete these questions even if the mother is not the primary caregiver for the enrolled child.* | | | |
| 1 | What is your age? | 10-99 *(years)* | ⬜ ⬜ |
| 2 | Are you currently married, divorced, widowed, or never married?  *If never married, skip to question 4.* | Never married = 01; Married = 02  Divorced = 03; Widowed = 04;  Separated = 05 | ⬜ ⬜ |
| 3 | How old were you when you got married for the first time? | 08-50 *(years)* | ⬜ ⬜ |
| 4 | Have you ever attended formal school?  *If no, skip to question 9.* | Yes = 01; No = 00 | ⬜ ⬜ |
| 5 | How many years of schooling have you completed? | 00-20 | ⬜ ⬜ |
| 6 | Have you ever received religious education (Madrasa)? | Yes = 01; No = 00 | ⬜ ⬜ |
| 7 | How many years of religious education have you completed? | 00-20 | ⬜ ⬜ |
| 8 | *If younger than 25 years old:* Are you currently attending school or college? | Yes = 01; No = 00 | ⬜ ⬜ |
| **FAMILY PLANNING QUESTIONS** | | | |
| 9 | How old were you when you first became pregnant? | 10-50 (years) | ⬜ ⬜ |
| 10 | How many pregnancies have you had in your lifetime? | 00-20 | ⬜ ⬜ |
| 11 | How many live births have you had in your lifetime? | 00-20 | ⬜ ⬜ |
| 12 | Are all of these children still alive? | Yes = 01; No = 00 | ⬜ ⬜ |
| 13 | How many children have died? | 00-20 | ⬜ ⬜ |
| 14 | What have you done to avoid pregnancy throughout your life? | Oral birth control pills = 01; Male Condoms = 02; Female Condoms = 03; Spermicide = 04; Abstinence = 05; Other = 05 | ⬜ ⬜ |
| 15 | If other, what type? |  | |
| 16 | How long did you use this method? | 00 – 999 months | ⬜ ⬜ |
| 17 | Have you had periods of time when you wanted to be pregnant but were not? | Yes = 01; No = 00 | ⬜ ⬜ |
| 18 | When were these periods? (e.g. between which pregnancies?) |  | |
| 19 | How long did each of these periods last? |  | |
| 20 | During these periods, were you ever concerned that you were not becoming pregnant? | Yes = 01; No = 00 | ⬜ ⬜ |
| 21 | What did you do to help become pregnant? |  | |
| 22 | How many children is a good number for a woman to have? | 00-20 | ⬜ ⬜ |
| 23 | What can a girl do to make sure that she is able to have healthy pregnancies and babies when she is a woman? |  | |
| 24 | What does a woman do to prepare for motherhood before pregnancy? |  | |
| 25 | What precautions during a pregnancy should a woman take to produce a healthy baby? |  | |
| 26 | Where are you planning to give birth the next time you are pregnant? |  | |
| 27 | Who would you like to attend the delivery with you? (sisters, mothers, etc?) |  | |
| 28 | Since [CHILD’S NAME] have you had another pregnancy? *If no skip to 30.* | Yes = 01; No = 00 | ⬜ ⬜ |
| 29 | How old is this most recently delivered child? | 00 – 36 months | ⬜ ⬜ |

| **SOCIO-ECONOMIC STATUS QUESTIONS** | | | |
| --- | --- | --- | --- |
| 30 | How long has your family lived in your house? | Less than one year = 01  Between one year and five years = 02  Between five years and ten years = 03  Between ten years and twenty years= 04  More than twenty years = 05 | ⬜ ⬜ |
| 31 | How many rooms are there in your house? | 01-15 | ⬜ ⬜ |
| 32 | How many rooms in this household are used for sleeping? | 01-10 (rooms) | ⬜ ⬜ |
| 33 | How many people usually sleep in this household? | 01-30 (people) | ⬜ ⬜ |
| 34 | Does your household pay any domestic workers? | Yes = 01; No = 00 | ⬜ ⬜ |
| 35 | What is the main source of drinking water for members of your household? | Piped into dwelling = 01  Piped to yard/plot = 02  Public tap/stand pipe= 03  Tube well or borehole = 04  Protected well = 05  Protected spring = 06  Rainwater collection = 07  Unprotected dug well = 08  Unprotected spring = 09  Cart with small tank or drum = 10  Tanker truck = 11 Surface water (river/ dam/ lake/pond/ stream/canal/irrigation canal) = 12  Bottled Water = 13 Other = 14 | ⬜ ⬜ |
| 36 | Is your piped water supply continuous or is it sometimes interrupted?  *If continuous, skip to question 38.* | Continuous = 01; Sometimes interrupted = 02 | ⬜ ⬜ |
| 37 | How long do these interruptions usually last? | Less than 3 hours = 01;  3 to 7 hours=02;  8 to 11 hours = 03;  12 to 24 hours= 04; More than 24 hours = 05 | ⬜ ⬜ |
| 38 | What is the main source of water used by your household for other purposes such as cooking and hand-washing? | Piped into dwelling = 01  Piped to yard/plot = 02  Public tap/stand pipe= 03  Tube well or borehole = 04  Protected well = 05  Protected spring = 06  Rainwater collection = 07  Unprotected dug well = 08  Unprotected spring = 09  Cart with small tank or drum = 10  Tanker truck = 11 Surface water (river/ dam/ lake/pond/ stream/canal/irrigation canal) = 12  Bottled Water = 13 Other = 14 | ⬜ ⬜ |
| 39 | Do you pay or barter for water? | Yes = 01; No = 00 | ⬜ ⬜ |
| 40 | Where is the water source located? | In own dwelling = 01;  In own yard/plot = 02; Elsewhere = 03 | ⬜ ⬜ |
| 41 | How long does it take to go there, get water and come back in one trip?  *If water is located on the premises, response is 000.* | 000-999 minutes | ⬜ ⬜ ⬜ |
| 42 | Who is the main person in the household who goes to fetch water from this source? | Adult woman = 01; Adult man = 02; Female child under age 15 years = 03; Male child under age 15 years = 04; Other = 05 | ⬜ ⬜ |
| 43 | Do you treat your water in any way to make it safer to drink? | Yes = 01  No = 00 | ⬜ ⬜ |
| 44 | What do you usually do to the water to make it safer to drink? | Let it stand and settle = 01  Solar disinfection = 02  Use water filter (ceramic /sand/ composite/etc.) = 03  Strain through a cloth = 04  Add bleach/chlorine = 05  Boil = 06  Other = 07 | ⬜ ⬜ |
| 45 | Do you wash your hands after helping your child defecate or changing his/her diaper? | Never = 01; Rarely = 02  Sometimes = 03; Always = 04 | ⬜ ⬜ |
| 46 | Do you wash your hands before preparing food? | Never = 01; Rarely = 02  Sometimes = 03; Always = 04 | ⬜ ⬜ |
| 47 | Do you wash your hands after using the toilet? | Never = 01; Rarely = 02  Sometimes = 03; Always = 04 | ⬜ ⬜ |
| 48 | Do you use toilet paper? | Never = 01; Rarely = 02  Sometimes = 03; Always = 04 | ⬜ ⬜ |
| 49 | What kind of toilet facility do members of your household usually use? | Flush/pour-flush to piped sewer system = 01  Flush/pour-flush to septic tank = 02  Flush/pour-flush to pit latrine = 03  Ventilated improved pit (VIP) latrine = 04  Pit latrine with slab = 05  Composting toilet = 06  Flush to somewhere else = 07  Pit latrine without slab/open pit = 08  Bucket = 09  Hanging toilet or hanging latrine = 10  No facility/bush/field or bucket toilet = 11  Other = 12 | ⬜ ⬜ |
| 50 | Do you share this toilet facility with other households? | Yes = 01  No = 00 | ⬜ ⬜ |
| 51 | How many households use this toilet facility? | 00-09; 10 or more households = 10 | ⬜ ⬜ |
| 52 | Does your household ever have electricity?  *If no, skip to question 55* | Yes = 01; No = 00 | ⬜ ⬜ |
| 53 | Is your electricity supply continuous year-round, or is it sometimes interrupted?  *If continuous, skip to question 55.* | Continuous = 01  Sometimes interrupted = 02 | ⬜ ⬜ |
| 54 | How long do these interruptions usually last? | Less than 3 hours = 01  3 to 7 hours = 02  8 to 11 hours = 03  12 to 24 hours = 04  More than 24 hours = 05 | ⬜ ⬜ |
| 55 | In case of discontinued power supply, what source does this household usually use? | Generator = 01; Gas/petrol = 02  UPS system = 03; Chargeable lights= 04  Other = 05, None = 06 | ⬜ ⬜ |
| *Now I am going to ask you about whether your household owns a series of items. Please respond yes if you own the item and it is in working form. If you own the item but it is broken or not working, please respond no.* | | | |
| 56 | Does your household have an iron (either charcoal or electric)? | Yes = 01; No = 00 | ⬜ ⬜ |
| 57 | Does your household have a mattress? | Yes = 01; No = 00 | ⬜ ⬜ |
| 58 | Does your household have a mat or bench? | Yes = 01; No = 00 | ⬜ ⬜ |
| 59 | Does your household have a sofa? | Yes = 01; No = 00 | ⬜ ⬜ |
| 60 | Does your household have a cupboard? *Includes cupboards with shutters and open shelves.* | Yes = 01; No = 00 | ⬜ ⬜ |
| 61 | Does your household have a table? | Yes = 01; No = 00 | ⬜ ⬜ |
| 62 | Does your household have an electric fan? | Yes = 01; No = 00 | ⬜ ⬜ |
| 63 | Does your household have a radio or transistor? | Yes = 01; No = 00 | ⬜ ⬜ |
| 64 | Does your household have a computer? | Yes = 01; No = 00 | ⬜ ⬜ |
| 65 | Does your household have a television? | Yes = 01; No = 00 | ⬜ ⬜ |
| 66 | Does your household have a mobile telephone? | Yes = 01; No = 00 | ⬜ ⬜ |
| 67 | Does your household have a refrigerator? | Yes = 01; No = 00 | ⬜ ⬜ |
| 68 | Does your household have a watch or clock? | Yes = 01; No = 00 | ⬜ ⬜ |
| 69 | Does you own a motorized vehicle (i.e. automobile, scooter)? | Yes = 01; No = 00 | ⬜ ⬜ |
| 70 | Does your household have a bicycle? | Yes = 01; No = 00 | ⬜ ⬜ |
| 71 | Does any member of your household have a bank account? | Yes = 01; No = 00 | ⬜ ⬜ |
| 72 | Does this household own any agricultural land? | Yes = 01; No = 00 | ⬜ ⬜ |
| 73 | Does your household own any coconut trees? | Yes = 01; No = 00 | ⬜ ⬜ |
| 74 | How much agricultural land does this household own? | 00-9999 (acres) |  |
| 75 | Does your household own cows or goats? | Yes = 01; No = 00 | ⬜ ⬜ |
| 76 | Does your household own chickens or ducks? | Yes = 01; No = 00 | ⬜ ⬜ |
| 77 | Do you ever cool your house?  *If no, skip to question 79.* | Yes = 01; No = 00 | ⬜ ⬜ |
| 78 | What is the primary source of fuel used for cooling in your household? | Electric Fan = 01; Air Conditioning Unit = 02; Other = 03 | ⬜ ⬜ |
| 79 | What type of cooking stove is mainly used in your house? | Kerosene stove = 01  Gas stove = 02; Open fire = 03  Open fire or stove with  chimney or hood = 04  Closed stove with chimney = 05  Electric heaters = 06; Other = 07 | ⬜ ⬜ |
| 80 | Is cooking done inside the house, outside the house, or both? | Inside the house = 01; Outside the house = 02; Both inside and outside the house = 03 | ⬜ ⬜ |
| 81 | What is the main material of the floor of your home? | Earth/sand/clay/mud/dung = 01;  Wood = 02;  Ceramic tiles or vinyl = 03;  Cement/concrete = 04;  Other = 05 | ⬜ ⬜ |
| 82 | What is the main material of the roof of your home? | No roof = 01  Thatch = 02  Metal = 03  Wood = 04  Brick = 05  Tiles = 06  Slate = 07  Other = 08 | ⬜ ⬜ |
| 83 | What is the main material of the exterior walls of your home? | No walls = 01  Mud = 02  Wood = 03  Cement/concrete = 04  Stone = 05  Metal = 06  Other = 07 | ⬜ ⬜ |
| 84 | What is the average monthly income *(in Kenyan shilling)* for the entire household? | ⬜ ⬜ ⬜ ⬜ ⬜ ⬜ ⬜ ⬜ | |
