# Supplementary material for: Factors associated with early childhood stunted growth in a 2012–2015 birth cohort monitored in the rural Msambweni area of coastal Kenya: a cross-sectional study
Source: BMC Pediatr. 2020 May 12;20:208. doi: 10.1186/s12887-020-02110-z (PMC7216696; doi:10.1186/s12887-020-02110-z)
Supplement: Supplementary file 2 — Additional File 2. Standardized mean difference of HAZ at 6-month intervals, by stunting status, for all study participants and for the subset of 77 study participants with complete anthropometric data from 6 to 24 months of age. [file 12887_2020_2110_MOESM2_ESM.docx]

**Supplemental Table 2, Additional File 2.** Mean HAZ at 6 month intervals for all study participants and for the subset of 77 study participants with complete anthropometric data from 6-24 months of age.

| Age | | HAZ (mean ± SD) | | | | p-value | | SMD | |
| --- | --- | --- | --- | --- | --- | --- | --- | --- | --- |
|  |  | Normal  (n=184 (75%)) | | Stunted  (n=60 (25%)) | |  |  |  |  |
| **N = 244** | |  | |  | |  | |  | |
| 6 month | | -0.50 ± 1.10 | | -1.74 ± 1.10 | | <0.001^a^ | | 1.129 | |
| 12 month | | -0.76 ± 0.92 | | -2.28 ± 0.98 | | <0.001^a^ | | 1.609 | |
| 18 month | | -1.02 ± 0.84 | | -2.25 ± 0.78 | | <0.001^a^ | | 1.505 | |
| 24 month | | -1.11 ± 0.86 | | -2.47 ± 0.75 | | <0.001^a^ | | 1.696 | |
| 30 month | | -1.20 ± 0.67 | | -2.32 ± 0.38 | | <0.001^a^ | | 2.071 | |
| 36 month | | -1.26 ± 0.30 | | -2.63 ± 0.35 | | 0.002^a^ | | 4.172 | |
|  |  | | | |  | |  | |  |
| Age | HAZ (mean ± SD) | | | | p-value | | SMD | |  |
|  | Normal  (n=63 (82%)) | | Stunted  (n=14 (18%)) | |  |  |  |  |  |
| **N = 77** |  | |  | |  | |  | |  |
| 6 month | -0.50 ± 1.10 | | -1.60 ± 1.24 | | 0.001^a^ | | 0.937 | |  |
| 12 month | -0.85 ± 1.01 | | -2.13 ± 0.69 | | <0.001^a^ | | 1.484 | |  |
| 18 month | -1.08 ± 0.80 | | -2.01 ± 0.81 | | <0.001^a^ | | 1.161 | |  |
| 24 month | -1.21 ± 0.63 | | -2.39 ± 0.77 | | <0.001^a^ | | 1.669 | |  |
|  | |  | |  | |  | |  | |
